# Longitudinal Assessment of Metabolic Syndrome and Direct-Acting Antiviral (DAA) Treatment Adherence in Hepatitis C Patients

**Published:** 2026-03-13

**Authors:** CHRISTA MATHEW, SAUD GUL, MANAHIL MONIS, SADIA BINTE TARIQ, AROOBA FNU, AIMEN JAMES, ABEEHA MAHMOOD, ZILL-E-RUKH FATIMA AMER, LOLOA RAED MOHAMMAD ALBSOUL, MOHAMMED HAKIM SHAH CHUN MOHAMMED, REUEL ABRAHAM KURUDAMANNIL, BILAL MOHAMMAD SHAFIQ, REEM ASHRAF FATHY ZALAT

**Affiliations:** 1 Department of Medicine, Royal Devon University Healthcare NHS Foundation Trust, Barnstaple, United Kingdom; 1 Department of Medicine, Royal Devon University Healthcare NHS Foundation Trust, Barnstaple, United Kingdom; 2 Department of Medicine, Royal Derby Hospital, Derby, United Kingdom; 2 Department of Medicine, Royal Derby Hospital, Derby, United Kingdom; 3 >Department of Medicine, Foundation University Medical College, Islamabad, Pakistan; 3 Department of Medicine, Foundation University Medical College, Islamabad, Pakistan; 4 Department of Medicine, Fatima Jinnah Medical University, Lahore, Pakistan; 4 Department of Medicine, Fatima Jinnah Medical University, Lahore, Pakistan; 5 Department of Medicine, People’s University of Medical & Health, Sciences for Women (PUMHSW), Nawabshah (SBA), Pakistan; 5 Department of Medicine, People’s University of Medical & Health Sciences for Women (PUMHSW), Nawabshah (SBA), Pakistan; 6 Department of Cardiology, Queen Elizabeth Hospital, King’s Lynn, United Kingdom; 6 Department of Cardiology, Queen Elizabeth Hospital, King’s Lynn, United Kingdom; 7 Department of Medicine, CMH Lahore Medical College, Lahore, Pakistan; 7 Department of Medicine, CMH Lahore Medical College, Lahore, Pakistan; 8 Department of Medicine, University of Badji Mokhtar Annaba, Annaba, Jordan; 8 Department of Medicine, University of Badji Mokhtar Annaba, Annaba, Jordan; 9 Department of Medicine, NMC Specialty Hospital, Abu Dhabi, United Arab Emirates; 9 Department of Medicine, NMC Specialty Hospital, Abu Dhabi, United Arab Emirates; 10 >Department of Medicine, Ternopil National Medical University, Ternopil, Ukraine; 10 Department of Medicine, Ternopil National Medical University, Ternopil, Ukraine

**Keywords:** hepatitis C, direct-acting antivirals (DAAs), metabolic syndrome, medication adherence, longitudinal study

## Abstract

**Objectives:**

Chronic hepatitis C is commonly associated with metabolic abnormalities that can influence adherence to direct-acting antiviral (DAA) therapy. This paper discussed the relationship between metabolic syndrome risk and compliance with DAA for 6 months.

**Materials and Methods:**

A longitudinal observational study was conducted among 402 patients with chronic hepatitis C taking DAA. Metabolic risk was measured using the Metabolic Screening Questionnaire (MSQ), and treatment adherence was measured at baseline, 3 months and 6 months using the Morisky Medication Adherence Scale (MMAS-8). Descriptive statistics, Spearman correlations, Mann-Whitney U test, Kruskal-Wallis tests and repeated-measures ANOVA were used as statistical tests.

**Results:**

Adherence at baseline (ρ = 0.28, p < 0.001), after 3 months (ρ = 0.22, p < 0.001), and after 6 months (ρ = 0.26, p < 0.001) was significantly associated with metabolic risk in a negative direction. Women adhered more than men (U = 16,950, p = 0.002), and men were more at risk of metabolism (U = 17,250, p = 0.008). MSQ (χ^2^ = 12.84, p = 0.012) and MMAS-8 (χ^2^ = 10.92, p =  0.028) differed in terms of age. Compliance at baseline (12.74) was compared with that at 6 months (13.62; F = 21.67, p < 0.001), and with metabolic risk being a significant mediator (F = 7.18, p < 0.001).

**Conclusion:**

Reduced treatment adherence to DAA therapy is closely associated with increased metabolic risk. Implementing metabolic risk management into standard care can enhance adherence and treatment outcomes among patients with hepatitis C.

## Introduction

Hepatitis C virus (HCV) is a blood-borne, hepatotropic RNA virus that leads to progressive liver disease; the most common genotypes are 1, 3, and 4, and direct-acting antivirals (DAAs) are highly effective for achieving viral clearance^1, 2)^. HCV is a significant cause of chronic liver disease, cirrhosis, hepatocellular carcinoma, and extrahepatic complications, affecting approximately 3% of the global population. Disease progression depends on host, viral, and environmental factors^3, 4)^.

HCV infection has been linked to extrahepatic systemic complications, such as cardiovascular, kidney, and metabolic complications. Genetic variants, such as PNPLA3 and TM6SF2, have been shown to influence the risk of metabolic complications and the severity of chronic kidney disease (CKD) in patients with HCV^5, 6)^. Metabolic imbalances such as insulin resistance, glucose and lipid regulation, and atherosclerosis are also associated with chronic HCV infection and not only influence chronic progression of liver disease, but also add to systemic extrahepatic manifestations^7)^.

DAAs have demonstrated the ability to clear HCV, improve both hepatic and extrahepatic outcomes, and reduce cardiovascular and metabolic risks. Nevertheless, more severe liver disease can increase the risk of hepatocellular carcinoma^8)^. Although IFN- free DAAs have dramatically improved outcomes, treatment adherence remains suboptimal (~52%), with lack of follow-up and limited disease awareness representing significant barriers, particularly among patients with hepatocellular carcinoma^9)^.

The interaction between metabolic syndrome and DAA compliance to achieve optimal HCV outcomes needs to be understood. Although metabolic complications and treatment adherence have been studied separately by other researchers, few longitudinal studies combine both measures. This type of research is critical for identifying patients at increased risk of worse outcomes and for informing specific interventions aimed at improving not only patients' metabolic status but also compliance with antiviral therapy.

This study aims to longitudinally assess the occurrence and progression of metabolic syndrome in patients receiving DAA therapy for HCV, while concurrently evaluating patterns of treatment adherence. By integrating these analyses, the study seeks to inform clinical decision-making and improve overall care for patients with HCV.

### Objectives

The purpose of the proposed research is to longitudinally evaluate the prevalence and progression of metabolic syndrome in patients with chronic hepatitis C receiving DAA treatment, as well as to assess longitudinal treatment adherence throughout. The research seeks to establish relationships between metabolic risk factors and adherence patterns, and to offer insights into factors that might determine the effectiveness of DAA treatment in people with HCV, and to guide strategies to maximise metabolic health and the overall efficacy of DAA treatment.

## Materials and Methods

### Study design and setting

This was a longitudinal observational study conducted between March 2025 and September 2025. The research aimed to evaluate metabolic syndrome and compliance with treatment in patients with chronic hepatitis C undergoing DAA treatment throughout the treatment.

### Study population

Individuals were eligible to participate in the study if they met the following criteria: patients aged 18 years or older with a known chronic hepatitis C infection who were receiving DAA therapy. The exclusion criteria were: co-infection with hepatitis B or HIV, decompensated liver disease, pregnancy, or a condition that might be a limiting factor to study follow-up or proper evaluation of metabolic parameters.

### Sample size and sampling technique

This study used convenience sampling to recruit 402 patients who were available and willing to participate throughout the study period. The sample size was estimated from an infinite target population, as no specific count of adults with hepatitis C in the study area was available. A 95% confidence level and a 5% margin of error were used, and a conservative estimate of 50% prevalence was employed to ensure the maximum sample size and account for population variation^10)^.

### Data collection tools

The data were collected at the baseline and the follow-up with the use of structured questionnaires, including:

**Demographic and clinical data:** Baseline data included age, gender, body mass index (BMI), lifestyle (diet, exercise, smoking), and comorbidities to characterise the study population.

** Metabolic screening questionnaire:** Metabolic health was assessed using a structured questionnaire that measured parameters needed to identify metabolic syndrome according to the International Diabetes Federation (IDF) 2006 criteria. The IDF consensus definition, developed by Alberti K.G.M., Zimmet P., Shaw J., et al., defines metabolic syndrome as central obesity and two of the following: elevated triglycerides, decreased HDL cholesterol, elevated blood pressure, or elevated fasting plasma glucose^11)^. The study employed this questionnaire to filter out possible metabolic risk factors and identify the existence of metabolic syndrome in patients with hepatitis C. It should be noted that while the MSQ was used to identify potential metabolic risk factors, biochemical confirmation (such as fasting glucose, lipid profile, or blood pressure measurements) was beyond the scope of this study.

** Morisky medication adherence scale (MMAS-8):** The 8-item Morisky Medication Adherence Scale (MMAS-8), which was developed by Donald Morisky in 2008, was used to assess adherence to DAA therapy. The MMAS-8 is a well-established self- report questionnaire that is used to evaluate medication-taking behaviour. It consists of 8 questions: 7 yes/no and one rated on a 5-point Likert scale. The overall scores range from 0 to 8, with higher scores indicating improved adherence. The compliance falls under high (score 8), medium (score 6 to < 8), and low (score < 6)^12)^. To identify patterns of DAA therapy adherence over time, longitudinal adherence was measured using the MMAS-8 at baseline, 3 months, and 6 months. For clarity, the mean MMAS-8 scores reported in the Results section reflect the average adherence level of all participants at each time point.

### Ethical considerations

The study protocol was approved by the ethical review committee of Queen Elizabeth Hospital (IRB: QEH-RD-ETHICS-25-1673). The participants in the study were informed about the study's purpose, methods, and potential risks and benefits. Oral informed consent was taken before data collection. The patient's confidentiality was maintained with great care throughout the study. Participation was voluntary, and patients could drop out at any time without affecting their treatment.

### Data analysis

SPSS version 26 was used to analyse the data. The demographic and clinical characteristics, i.e., metabolic risk and medication adherence, were summarised using descriptive statistics (frequencies, percentages, and mean ranks where appropriate). The relationship between baseline, 3-month, and 6- month metabolic risk (MSQ) and medication adherence (MMAS-8) was examined using Spearman's rank-order correlation. Metabolic risk and compliance were compared across gender and age groups using the Mann-Whitney U test and the Kruskal- Wallis test, respectively. A repeated-measures ANOVA was used to assess longitudinal variation in medication adherence over time and to examine the interaction between medication adherence and metabolic risk. Any p-value below 0.05 was deemed statistically significant.

### Demographic and clinical characteristics of the study participants

The demographic and clinical profile of 402 participants is characterised in Table 1. The majority were aged 50-59 years (48.8%), with a slightly larger proportion of women (51.5%). Marital status varied, with the most significant subgroups being divorced or separated (29.6%), married (25.9%), and single (24.4%). Educational levels varied, but almost half had no formal education or only a primary education. Employment was evenly distributed: 31.1% of respondents were employed, with the rest being students, unemployed, or retirees. The BMI categories showed that the majority consisted of underweight (31.6%) participants, followed by 24.1% overweight and obese (20.6%). The prevalence of smoking was high, with 44.5% being current smokers. A large percentage of respondents had chronic conditions, such as diabetes (28.4%) and high blood pressure (27.9%). Over 50% had a history of 4 or more years of HCV diagnosis, and a considerable percentage (44.0%) of those infected had undergone treatment with interferon-based regimens, with 39.8% being treatment-naive.

**Table 1:** Demographic characteristics of participants (N = 402)

Variable	f	%		Variable	f	%
Age				Body mass index (BMI)		
18-29 years	11	2.7		Underweight (< 18.5)	127	31.6
30-39 years	56	13.9		Normal weight (18.5-24.9)	95	23.6
40-49 years	103	25.6		Overweight (25-29.9)	97	24.1
50-59 years	196	48.8		Obese (≥ 30)	83	20.6
60 years and above	36	9.0		Smoking status		
Gender				Never smoked	93	23.1
Male	195	48.5		Former smoker	130	32.3
Female	207	51.5		Current smoker	179	44.5
Marital status				History of chronic conditions	175	58.3
Single	98	24.4		Hypertension	112	27.9
Married	104	25.9		Diabetes mellitus	114	28.4
Divorced/Separated	119	29.6		Hyperlipidemia	73	18.2
Widowed	81	20.1		Cardiovascular disease	59	14.7
Educational level				None of the above	44	10.9
No formal education	89	22.1		Duration since HCV diagnosis		
Primary school	90	22.4		< 1 year	107	26.6
Secondary school/High school	95	23.6		1-3 years	73	18.2
Graduate (Bachelor's)	85	21.1		4-6 years	128	31.8
Postgraduate (Master's or above)	43	10.7		> 6 years	94	23.4
Employment status				Previous HCV treatment history		
Student	86	21.4		Treatment-naïve	160	39.8
Employed	125	31.1		Previously treated with interferon-based therapy	177	44.0
Unemployed	90	22.4		Previously treated with DAAs	65	16.2
Retired	101	25.1				

Note. N = number of participants; f = frequency, % = percentage

### Correlation between metabolic risk and medication adherence across follow-up periods

Table 2 indicates a significant correlation between metabolic risk and medication adherence at all time points. Increased scores of metabolic risk were consistently correlated with reduced baseline, 3 months, and 6 months medication adherence (0.22 - 0.28, all p < 0.001). Baseline medication adherence scores were positively associated with adherence at 3 and 6 months, suggesting consistent adherence over time. On the same note, compliance at 3 months was highly associated with compliance at 6 months. Overall, the findings indicate that greater metabolic risk is associated with poorer adherence, but there is no significant difference in adherence behaviours across follow-up times.

**Table 2:** Spearman's rank-order correlations between metabolic risk and medication adherence across time points (N = 402)

Variable	Metabolic screening questionnaire (MSQ)	Morisky medication adherence scale (Baseline)	Morisky medication adherence scale (3 months)	Morisky medication adherence scale (6 months)
Metabolic screening questionnaire (MSQ)	-	ρ = -0.28, t = -5.88, p = < 0.001***	ρ = -0.22, t = -4.52, p = < 0.001***	ρ = -0.26, t = -5.43, p = < 0.001***
Morisky medication adherence scale (Baseline)	-	-	ρ = 0.34, t = 7.27, p = < 0.001***	ρ = 0.41, t = 9.01, p = < 0.001***
Morisky medication adherence scale (3 months)	-	-	-	ρ = 0.38, t = 8.23, p = < 0.001***
Morisky medication adherence scale (6 months)	-	-	-	-

Note. ***= *p* < 0.001 considered significant; Positive correlations indicate direct relationships, and negative correlations indicate inverse relationships; N = 402.

### Gender differences in metabolic risk and medication adherence

According to Table 3, there is a significant difference between genders in terms of metabolic risk and medication adherence. The metabolic risk scores were higher in men than in women, with a significantly higher mean rank (215.60 vs. 187.20; U = 17,250, p = 0.008). Women, in contrast, showed much higher medication adherence than men (mean rank 216.00 vs. 185.50; U = 16,950, p = 0.002). These results indicate that males in the sample were more at risk of metabolic disorders, and females were more likely to take medication.

**Table 3:** Gender differences in metabolic risk and medication adherence based on Mann-Whitney U tests (N = 402)

Variable	Gender	N	Mean rank	Sum of ranks	U	Z	p
Metabolic screening questionnaire (MSQ)	Male	195	215.60	42,042.00	-	-	-
	Female	207	187.20	38,750.40	17,250	-2.65	0.008**
Morisky medication adherence scale (MMAS-8)	Male	195	185.50	36,172.50	-	-	-
	Female	207	216.00	44,712.00	16,950	-3.05	0.002**

Note. N = 402 (Males = 195, 48.5%; Females = 207, 51.5%); Mann-Whitney U test was used for all comparisons; p values marked with ** indicate statistical significance at p < 0.01

### Age-related differences in metabolic risk and medication adherence

Table 4 shows a significant difference in metabolic risk and medication adherence between the age groups. The metabolic risk (MSQ scores) showed considerable variability, with the highest mean rank observed in the 30-39 and 40-49 age groups, and the lowest rank observed in the youngest (18-29) and the oldest (60+) participants (χ^2^ = 12.84, p = 0.012). Age also significantly differed in medication adherence (MMAS-8 scores), with the youngest and the oldest (ages 40 and above, respectively) having the lowest levels of adherence (χ^2^(4) = 10.92, p = 0.028). Overall, the results show that metabolic risk levels and adherence change with age, peaking in middle adulthood and declining thereafter.

**Table 4:** Differences in metabolic risk and medication adherence across age groups based on Kruskal-Wallis tests (N = 402)

Variable	Age group	N	Mean rank	x^2^(df = 4)	p
Metabolic screening questionnaire (MSQ)	18-29 years	11	150.00	-	-
	30-39 years	56	225.00	-	-
	40-49 years	103	230.50	-	-
	50-59 years	196	215.80	-	-
	60 years or above	36	180.40	12.84	0.012*
Morisky medication adherence scale (MMAS-8)	18-29 years	11	160.00	-	-
	30-39 years	56	210.90	-	-
	40-49 years	103	215.30	-	-
	50-59 years	196	220.80	-	-
	60 years or above	36	175.20	10.92	0.028*

Note. N = number of participants in each age category; % = percentage of the total sample; Percentages are based on total N = 402; Values are mean ranks from Kruskal-Wallis H tests; Overall test statistics are reported in the bottom row for each dependent variable: Total MSQ (χ^2^(4) = 12.84, 0.012*) and Total MMAS-8 (χ^2^(4) = 10.92, 0.028*); Significance levels: p < 0.05*

### Changes in medication adherence over time and interaction with metabolic risk

Table 5 shows a considerable difference in medication adherence, with MMAS-8 scores higher at baseline (M = 12.74), 3 months (M = 13.29), and 6 months (M = 13.62). Repeated-measures ANOVA revealed a significant main effect of time (F = 21.67, p < 0.001, partial η 2 = 0.097), indicating that adherence increased significantly over time. The time × metabolic risk interaction was also significant (F = 7.18, p < 0.001, partial η 2 = 0.034), indicating that adherence varied across levels of metabolic risk. The total compliance levels increased gradually over time, and metabolic risk influenced these variations.

**Table 5:** Repeated measures ANOVA for changes in medication adherence (MMAS-8) over time and interaction with metabolic risk (MSQ)

Time Point	Mean	SD	N	-	-	-
Morisky medication adherence scale (baseline)	12.74	1.45	402	-	-	-
Morisky medication adherence scale (3 months)	13.29	1.52	402	-	-	-
Morisky medication adherence scale (6 months)	13.62	1.53	402	-	-	-
Source	Type III SS	df	Mean square	F	p	Partial η^2^
Time	18.45	2	9.225	21.67	<0.001***	0.097
Time × total metabolic screening questionnaire (MSQ)	6.12	2	3.060	7.18	<0.001***	0.034
Error(time)	341.75	800	0.427	—	—	—

Note. ***p < 0.001. Partial η^2^ represents effect size.

## Discussion

This longitudinal study examined the relationship between metabolic syndrome risk and treatment adherence in chronic hepatitis C patients receiving DAA therapy. The findings indicate that metabolic risk is closely correlated with medication adherence, with higher metabolic risk consistently associated with lower adherence at baseline, 3 months, and 6 months. These findings are consistent with previous studies among patients with metabolic syndrome, where nearly 43% of the sample reported low medication adherence, particularly among patients with high disease burden and complex treatment schedules^13)^. These results indicate that an integrated approach to care, including metabolic well-being and antiviral therapy, is necessary.

A gender difference was also observed in our study, with males having a higher metabolic risk than females. This is unlike in some population- based studies, which revealed that the prevalence rate of metabolic syndrome is higher in women. Still, it reminds us that gender-specific metabolic patterns might vary across populations^14)^. MMAS-8 scores indicated higher medication adherence among females compared to males. This finding runs counter to a wide range of claims-based research reporting lower adherence among women, and it provides evidence that adherence patterns are not universal in clinical and population-based settings^15)^.

We observed greater metabolic risk in middle-aged participants (30-49 years), consistent with a previous study reporting the highest incidence of metabolic syndrome among men aged 40-49 years, suggesting that midlife is the critical phase for accumulating metabolic risk^16)^. Interestingly, younger and older adults also had lower adherence levels, although middle-aged participants were the most adherent. This is, in part, in line with prior research that has found higher non-adherence among younger adults than among older adults, with the implication that adherence increases with age as individuals age into middle age^17)^.

Similar to our finding of increased adherence at 6 months (MMAS-8: 12.74, 13.62), previous longitudinal studies have reported improvement in medication adherence at that time as well, highlighting the importance of continued patient support and beliefs in adherence^18)^. In addition, it has been previously determined that worse compliance is associated with a higher level of metabolic risk factors, such as central obesity. Our study expands on this information by demonstrating that metabolic risk predicts not only reduced adherence but also influences adherence dynamics^19)^.

The overall findings of these studies indicate that multifaceted management methods that integrate assessment of metabolic risks, patient education, and adherence support can improve clinical outcomes for patients receiving antiviral therapy. Interventions targeting metabolic risk, such as lifestyle modification, dietary counselling, and structured physical activity programs, may indirectly enhance DAA adherence by improving patients’ overall health, motivation, and engagement with treatment. Moreover, tailored patient education and regular follow- up can empower individuals to manage metabolic risk factors better and improve adherence to antiviral therapy, ultimately leading to better long-term outcomes.

### Limitations and recommendations

This research has several limitations. Self-reported questionnaires (MMAS-8 and MSQ) and convenience sampling could limit generalisability and introduce bias. The assessment of metabolic risk was not performed, and the verification of biochemical factors and clinical outcomes, such as SVR or changes in liver function, was not analysed. Possible confounders (depression or socioeconomic factors) were not accounted for, and the follow-up period was only 6 months, which may not reflect long-term outcomes.

Future research should include biochemical markers of metabolic syndrome, larger probability- based samples, and clinical outcome measures. Psychosocial predictors and intervention strategies, including counselling or digital reminders, must also be sought to enhance adherence. Follow-up periods and gender- or age-specific strategy can be extended to understand adherence patterns better.

## Conclusion

The research indicates that there is a strong correlation between treatment adherence and metabolic syndrome risk amongst chronic hepatitis C patients receiving DAA therapy, with better adherence rates related to increased metabolic risk, and the reverse also being true. Gender and age were significant variables affecting both metabolic risk and compliance patterns. The findings suggest that there should be integrated clinical strategies for metabolic health and adherence to behaviour. Early identification of patients at high metabolic and adherence risk throughout treatment would enable medical professionals to implement targeted measures to improve antiviral therapy response and health outcomes.

## Data availability

The datasets generated and analyzed during the current study are available from the corresponding author on reasonable request.

## Author contributions

CM conceived and designed the study and supervised the overall project. SG contributed to the study design and clinical interpretation of metabolic outcomes. MM assisted in data acquisition and patient-related assessments. SBT contributed to patient recruitment and the collection of clinical information. AF supported data collection and helped with preliminary analysis. AJ served as the corresponding author, contributed to methodological development, and supervised statistical planning. AM participated in data entry and ensured data quality. ZRFA assisted with literature review and background drafting. LRMA contributed to the interpretation of clinical data and supported manuscript revisions. MHSCM assisted in managing hospital records and contributed to refining the Methods section. RAK supported data management and formatting of tables and figures. BMS contributed to the statistical analysis and the drafting of the results. RAFZ participated in proofreading, literature review, and critical revision of the manuscript. All authors read and approved the final manuscript.

## Conflicts of interest statement

The authors declare that there are no conflicts of interest.
